# Low-dose peripheral blood stem cell graft after high-dose chemotherapy - an evaluation of hematopoietic reconstitution

**DOI:** 10.1186/s12885-020-06873-7

**Published:** 2020-04-25

**Authors:** Sandra Sauer, Petra Pavel, Anita Schmitt, Martin Cremer, Mark Kriegsmann, Thomas Bruckner, Karin Jordan, Patrick Wuchter, Carsten Müller-Tidow, Katharina Kriegsmann

**Affiliations:** 1 Department of Hematology, Oncology and Rheumatology, Heidelberg University, Im Neuenheimer Feld 410, 69120 Heidelberg, Germany; 2 Stem Cell Laboratory, IKTZ Heidelberg GmbH, Heidelberg, Germany; 3 Institute of Pathology, Heidelberg University, Heidelberg, Germany; 4 Institute of Medical Biometry and Informatics, Heidelberg University, Heidelberg, Germany; 5 Institute of Transfusion Medicine and Immunology, Medical Faculty Mannheim, Heidelberg University, German Red Cross Blood Service Baden-Württemberg – Hessen, Mannheim, Germany

**Keywords:** Peripheral blood stem cells, Insufficient graft, Autologous stem-cell transplantation, Multiple myeloma

## Abstract

**Background:**

High-dose (HD) chemotherapy followed by autologous blood stem-cell transplantation (ASCT) is the standard treatment for multiple myeloma (MM) patients. However, the collection of sufficient peripheral blood stem cell (PBSC) grafts can be challenging, and the question arises whether reinfusion of low-dose grafts will lead to a hematopoietic recovery.

**Methods:**

The hematopoietic recovery of 148 MM patients who underwent HD melphalan chemotherapy and received PBSC transplants with varying CD34+ cells doses (3–4 × 10^6^ [*n* = 86], 2–2.5 × 10^6^ [*n* = 53], < 2 × 10^6^ [*n* = 9] per kg body weight [bw]) was analyzed in this retrospective single-center study.

**Results:**

All patients reached hematopoietic reconstitution, even those who received < 2 × 10^6^ CD34+ cells/kg bw. 62 (42%) patients received granulocyte-colony-stimulating factor (G-CSF). The median duration to leukocyte recovery ≥1.0 × 10^9^/L was 12 days in every group. The median duration to platelet recovery ≥20 × 10^9^/L was 11, 13 and 13 days, respectively. In the multivariate analysis, a low number of reinfused CD34+ cells was associated with prolonged time until leukocyte reconstitution (*p* = 0.010, HR 0.607) and platelet recovery (*p* < 0.001, HR 0.438). G-CSF support significantly accelerated leukocyte (p < 0.001, HR 16.742) but not platelet reconstitution.

**Conclusion:**

In conclusion, reinfusion of low- and even very-low-dose PBSC grafts leads to sufficient hematopoietic reconstitution. No severe adverse events were observed during or after HD chemotherapy and ASCT in the analyzed cohort. While the impact of CD34+ cell dose is marginal, G-CSF significantly accelerates the leukocyte recovery.

## Background

High-dose (HD) chemotherapy followed by autologous blood stem-cell transplantation (ASCT) is the standard of care and a highly effective therapy for multiple myeloma (MM) [1, 2]. Although HD/ASCT was initially established as a single therapy for first-line treatment of MM [3, 4], subsequent randomized trials demonstrated an overall survival benefit with tandem ASCT, particularly in patients who did not achieve at least partial remission (PR) [5, 6]. Later studies showed that salvage HD/ASCT may represent an effective treatment option for MM patients who relapse after a sustained remission that lasted longer than 1 year after a prior ASCT [7–9]. The indication for up to three HD/ASCTs might occur over the course of MM treatment.

As a prerequisite for ASCT, hematopoietic stem cells must be available. Peripheral blood stem cells (PBSCs) have become the most widely used source for hematopoietic stem cells in the setting of HD/ASCT treatment for MM [10, 11]. PBSCs (i.e., CD34+ cells) must be mobilized either with mobilization chemotherapy and granulocyte-colony-stimulating factor (G-CSF) or with G-CSF alone and subsequently collected by leukapheresis [12]. Usually, a successful collection of up to three sufficient PBSC grafts (> 2.0–2.5 × 10^6^ CD34+ cells/kg body weight [bw] per graft) from MM patients can be achieved when the PBSC collection is performed after induction treatment i.e., prior to the first HD/ASCT [13, 14]. However, many factors, such as higher age, previous extensive chemotherapy, and treatment with melphalan or radiation therapy, might be associated with poor PBSC mobilization, despite the use of plerixafor, which results in borderline sufficient (< 2.0–2.5 × 10^6^ CD34+ cells/kg bw) grafts [15–19]. In this case, transplant centers frequently face the question of whether reinfusion of grafts with marginal PBSC numbers will lead to a delay in hematopoietic recovery after HD chemotherapy and subsequently cause any complications or even severe adverse events due to prolonged neutropenia. This issue is of great relevance, particularly to MM patients who might significantly benefit from HD/ASCT treatment in terms of MM disease control.

The aim of this study was to demonstrate that hematopoietic reconstitution is not significantly delayed, even if a low (2.0–2.5 × 10^6^/kg bw) or a very low (< 2.0 × 10^6^/kg bw) number of PBSCs is reinfused during ASCT. Moreover, the question of whether the number of reinfused PBSCs affects the duration until achieving hematopoietic recovery will be answered.

## Methods

### Patient selection and data matching

A retrospective single-center analysis of MM patients who underwent HD melphalan chemotherapy and ASCT between January 2016 and August 2018 at our university hospital was performed. The patients were grouped according to the number of reinfused CD34+ cells at ASCT, as follows: 3–4 × 10^6^ CD34+ cells/kg bw (group 1), 2–2.5 × 10^6^ CD34+ cells/kg bw (group 2), < 2 × 10^6^ CD34+ cells/kg bw (group 3). Group 1 reflects the median reference value of reinfused CD34+ cells at our institution, as previously reported [20, 21]. To achieve homogenization between groups 1 and 2, only patients who received one round of HD/ASCT therapy in their course of treatment were included. As there were only few patients in group 3, the second or third HD/ASCT was also considered in this group. Patients in group 3 received in median 1,89 × 10^6^ (range 1,74 to 1,99 × 10^6^) CD34+ cells/kg bw. The clinical parameters (sex and age), ISS stage and Salmon and Durie stage at first diagnosis, type of monoclonal protein, modality of induction and mobilization therapy, remission status before and after each ASCT, number of transplanted CD34+ cells and hematological reconstitution data were collected retrospectively. The retrospective data analysis was approved by the Ethics Committee of the Medical Faculty, Heidelberg University.

### Multiple myeloma induction therapy

MM treatment was initiated according to the SLiM-CRAB criteria [22]. The standard induction treatment was 4 cycles of VCD (bortezomib 1.3 mg/m^2^, s.c., days 1, 4, 8, 11; cyclophosphamide 1000 mg/m^2^, i.v., day 1; dexamethasone 40 mg, p.o., days 1, 2, 4, 5, 8, 9, 11, 12). Sixty-three patients received either 4 cycles of VRD (bortezomib 1.3 mg/m^2^, s.c., days 1, 4, 8, 11; lenalidomide 25 mg, p.o., days 1–14; dexamethasone 20 mg, p.o., days 1, 2, 4, 5, 8, 9, 11, 12, 15 optional) or elotuzumab (10 mg/kg, i.v., days 1, 8, 15 in cycle 1 and 2; days 1, 11 in cycles 3 and 4) in combination with VRD as induction therapy. The remission status was assessed according to international myeloma working group response criteria [23].

### PBSC mobilization, collection and quality assessment

PBSC mobilization was performed as previously described [20]. In summary, CAD (cyclophosphamide 1000 mg/m^2^, i.v., day 1; doxorubicin 15 mg/m^2^, i.v., days 1–4; dexamethasone 40 mg, p.o., days 1–4) was administered as a standard chemomobilization regimen. Three patients received cyclophosphamide (1000 mg/m^2^/day, i.v., days 1–2) only. G-CSF (5–10 μg/kg per day) was injected subcutaneously starting 5 days after mobilization chemotherapy and was administered until the end of PBSC collection. The number of CD34+ cells was determined by flow cytometry as described previously when peripheral blood leukocytes reached ≥5.0 × 10^3^/μl [24]. When the peripheral blood CD34+ cell count reached ≥20/μl, leukapheresis (LP) was initiated. Stem cell collection was performed at the Spectra Optia apheresis machine (MNC program, software version 7.2 and 11.2). In the case of poor mobilization (i.e. < 20 CD34^+^ cells/μL under G-CSF stimulation or less than one third of the individual collection goal reached with the first leukapheresis session), pre-emptive or rescue plerixafor (240 μg/kg) was administered subcutaneously 9 to 12 h before the LP session. The minimum number of CD34+ cells for one transplant was defined as ≥2.0 × 10^6^/kg bw at our institution, with the goal of collecting sufficient CD34+ cells for three transplants to ensure the option for a tandem transplantation or anHD melphalan and ASCT in case of relapse.

PBSCs processing and storage was in accordance with the German Medical Council and further scientific society’s guidelines [25–27]. The PBSCs were stored for 24–48 h at 2 to 6 °C until cryopreservation. The maximum nucleated cell (NC) concentration was 2 × 10^8^/mL. After storage, the PBSC products were centrifuged and diluted with autologous plasma or resuspension medium (Plasmalyte A, Baxter, Unterschleissheim, Germany or Composol PS, Fresenius Kabi, Bad Homburg, Germany) and CryoSure-D dimethyl sulfoxide (DMSO, WAK-Chemie Medical, Steinbach, Germany). The target NC concentration was ≤5 × 10^8^/mL and the total volume was 100 mL per bag. The final product included 10% DMSO and was stored in Cryocyte bags (Baxter, Unterschleissheim, Germany or CryoMACS Freezing bags (Miltenyi, Idarobrstein, Germany). The PBSCs controlled-rate freezed (Biofreeze BV50, Consarctic, Schoellkrippen, Germany). The storage conditions were vapor-phase nitrogen and a temperature of <− 140 °C. Upon transplantation, the cryopreserved bags were thawed at the bedside (Plasmatherm device, Barkey GmbH & Co. KG, Leopoldshoehe, Germany) at 37 °C. PBSCs were reinfused without previous washing within a maximum of 10 min of thawing using standard transfusion filters.

For quality assessment in accordance with the Stem Cell Enumeration Committee Guidelines of the International Society for Cell Transplantation, a an enumeration of NC and red blood cells, flow cytometry-based CD34+ cell quantification and volume determination were performed directly after PBSC collection [28]. A microbiological culture sample was obtained shortly before freezing. NC enumeration and NC viability measurements were performed in the PBSC aliquots 48 h after freezing and in samples that were stored for a duration > 36 months. Overall, the following target values were defined for the end product (one PBSC transplant): NC concentration ≤ 5 × 10^8^/mL, CD34+ cell number ≥ 2 × 10^6^/kg bw, a total volume of 100 mL per portion (up to 3 portions possible), no microbial growth, and a minimum NC viability of 50%. Viability testing was valid for a maximum duration of 3 years.

### HD chemotherapy and ASCT

All patients received melphalan (100 mg/m^2^, day − 3 and day − 2, one-hour infusion) as high dose chemotherapy conditioningregimen. When creatinine clearance was ≤40 mL/min, the melphalan dosage was reduced by 50%.. An supportive medication regimen (dexamethasone 4 mg p.o., day − 3; dexamethasone 2 mg p.o., day − 2 to day − 1, granisetron hydrochloride 2 mg p.o., days − 3 to day 0, aprepitant 125 mg p.o., day − 3, aprepitant 80 mg p.o., day − 2 to day 0) was used for prevention of chemotherapy-induced nausea and vomiting [29]. A minimum of 2.0 × 10^6^ C34+ cells/kg bw was reinfused using supportive therapy (500 mg acetaminophen p.o., 2 mg clemastine i.v., 10 mg dihydrocodeine p.o.) on day 0. As antiviral and antibiotic prophylaxis, patients received daily acyclovir 2 × 400 mg p.o. for 6 months, dayli ciprofloxacin 2 × 500 mg p.o. until hematological reconstitution, and cotrimoxazole 960 mg p.o. three times a week for 3 months.

Our analysis comprises MM patients who underwent HD melphalan chemotherapy and ASCT between January 2016 and August 2018 at our university hospital. At our institution antibiotic prophylaxis with ciprofloxacin or cotrimoxazole twice a day was stopped in January 2017 due to increasing prevalence of multidrug resistant bacteria and replaced by G-CSF support after ASCT and *Pneumocystis jirovecii* pneumonia prophylaxis with cotrimoxazole thrice a week in March 2017. Therefore, in a subset of patients, G-CSF (10 μg/kg bw per day) was administered starting from day 1 after ASCT until leukocyte recovery ≥1.0 × 10^9^/L.

### Assessment of hematological reconstitution

After HD melphalan and ASCT, blood counts were performed dayli until leukocyte and platelet engraftment. Leukocyte engraftment was defined by a leukocyte count of ≥1.0 × 10^9^/L. Days in aplasia were defined as number of days with leukocytes < 1.0 × 10^9^/L. Neutrophil recovery was defined as the first of three consecutive days with neutrophils ≥0.5 × 10^9^/L. Platelet engraftment was defined as the first day of three consecutive values with platelet count ≥20 × 10^9^/L without previous platelet transfusion for 7 days. We also calculated days until the platelet count ≥50 × 10^9^/L as a variable for platelet engraftment, as the platelet count in some patients did not drop below 20 × 10^9^/L or was not assessable due to platelet transfusion.

### Statistical analysis

Statistical analysis was performed for the overall cohort and with regard to the number of reinfused CD34+ cells at ASCT. Due to the low number of patients in group 3, comparative statistics were performed between groups 1 and 2.

Descriptive statistics and comparisons between groups were performed by R studio (Version 1.1.383, RStudio, Inc.). Data are presented as absolute numbers and percentages and as medians and ranges. To compare categorical variables, the chi-square test was used. To identify differences between group means, comparisons between the two groups were performed with unpaired two-tailed Student’s t-tests. The leukocyte, neutrophil and platelet recovery over time was calculated and plotted using Kaplan-Meier survival analysis. To calculate differences between the engraftment curves, a log-rank test was applied. The Cox proportional hazard model and the Breslow method were used for multivariate analysis. A *p* < 0.05 was considered statistically significant.

## Results

### Patient characteristics

Data from 148 MM patients (87 male and 61 female) were analyzed. The median age at first diagnosis was 60 (41–72) years. International Staging System (ISS) stage I was found in 85 (57%), ISS II in 25 (17%), and ISS III in 31 (21%) patients, in 7 patients ISS stage was not available. In patients with stage I and II disease (*n* = 11) according to the Salmon-Durie classification at first diagnosis, the indications for treatment initiation were based on the SLiM CRAB criteria and were abnormal kappa/lambda ratio/involved free light-chain level 100 mg/L or higher (*n* = 8), bone marrow infiltration by plasma cells above 60% (*n* = 1) and more than one focal lesion on magnetic resonance imaging (*n* = 2). The majority of patients (*n* = 70, 47%) received VCD for induction treatment. Patients who were treated within the GMMG HD6 trial received either VRD (*n* = 30, 20%) or elotuzumab-VRD (*n* = 33, 22%). The median number of induction treatment cycles was 4 (range 2–8). Nearly all patients (*n* = 143, 97%) received CAD/G-CSF for PBSC mobilization. To achieve the PBSC collection goal, plerixafor administration was necessary in 2 (1%) patients.

Table 1 presents patient characteristics at first diagnosis and induction and mobilization therapy with regard to the overall cohort and subgroups defined by the number of transplanted CD34+ cells.

**Table 1:** Patient characteristics and previous therapy regimens

Parameter	Overall cohort	Group1 (3–4 × 10^**6**^ CD34+ cells/kg bw)	Group 2 (2–2.5 × 10^**6**^ CD34+ cells /kg bw)	***P*** value Group 1 vs. 2	Group 3 (< 2 × 10^**6**^ CD34+ cells /kg bw)
**Patient number, n**	148	86	53	/	9
**Sex, n (%)**				**0.030**	
Male	87 (59)	44 (51)	37 (70)		6 (67)
Female	61 (41)	42 (49)	16 (30)		3 (33)
**Diagnosis of MM, n (%)**					
Median age at first diagnosis, years (range)	60 (41–72)	60 (44–72)	61 (41–71)	0.854	60 (46–72)
Stage at first diagnosis				/	
I	7 (5)	4 (5)	3 (6)		9 (100)
II	4 (3)	3 (3)	1 (2)		0 (0)
III	136 (92)	79 (92)	48 (91)		0 (0)
NA	1 (1)	0 (0)	1 (2)		0 (0)
A	129 (87)	78 (91)	44 (83)	/	7 (78)
B	18 (12)	8 (9)	8 (15)		2 (22)
NA	1 (1)	0 (0)	1 (2)		0 (0)
Heavy chain type				0.767^a^	
IgG	95 (64)	56 (65)	37 (70)		2 (22)
IgA	29 (20)	17 (20)	8 (15)		4 (44)
IgD	1 (1)	1 (1)	0 (0)		0 (0)
Light chain only	23 (16)	12 (14)	8 (15)		3 (33)
Light chain type				0.452	
kappa	96 (65)	53 (62)	36 (68)		7 (78)
lambda	52 (35)	33 (38)	17 (32)		2 (22)
**Induction therapy, n (%)**					
Median number of cycles (range)	4 (2–8)	4 (2–6)	4 (3–8)		4 (3–5)
VCD	70 (47)	39 (45)	28 (53)	0.297^b^	3 (33)
VRD	30 (20)	22 (26)	8 (15)		0 (0)
Elotuzumab-VRd	33 (22)	22 (26)	10 (19)		1 (11)
Other/modifications	15 (10)	3 (3)	7 (13)		5 (56)
**Mobilization therapy, n (%)**				/	
1xCAD	143 (97)	85 (99)	50 (94)		8 (89)
Other	5 (3)	1 (1)	3 (6)		1 (11)
**Remission prior PBSC collection, n (%)**					
nCR	25 (17)	19 (22)	5 (9)	**0.041**^**c**^	1 (11)
VGPR	52 (35)	34 (40)	17 (32)		1 (11)
PR	54 (36)	25 (29)	25 (47)		4 (44)
MR	8 (5)	5 (6)	2 (4)		1 (11)
SD	1 (1)	1 (1)	0 (0)		0 (0)
NA	8 (5)	2 (2)	4 (8)		2 (22)

^a^IgD not included

^b^Other/modifications not included

^c^nCR/VGPR versus PR/MR/SD

*CAD* cyclophosphamide, doxorubicin, dexamethasone; *MM* multiple myeloma; *MR* minimal response; *NA* not available; *nCR* near complete remission; *PBSC* peripheral blood stem cells; *PR* partial remission; *SD* stable disease; *VCD* bortezomib, *VGPR* very good partial remission; *VRD(d)* vincristine, lenalidomide (revlimid), dexamethasone; cyclophosphamide, dexamethasone; vs., versus

### Characterization of HD/ASCT treatment according to the number of transplanted CD34+ cells

To answer the clinically important question whether the number of transplanted CD34+ cells impacts hematopoietic reconstitution after HD/ASCT therapy and achieving homogenization, we focused on the first HD/ASCT therapy in the patient’s course of treatment (groups 1 and 2). Fifty-three of the patients had a low dose graft (2–2.5 × 10^6^ CD34+ cells/kg) and three of the patients had a very low dose graft (< 2 × 10^6^ CD34+ cells/kg) for their first autologous transplant. However, reinfusion of < 2 × 10^6^ CD34+ cells/kg at ASCT was a rare event. Therefore, patients undergoing second or third HD/ASCT treatment were included in group 3.

In the overall cohort, 88 (59%) patients had complete remission (CR), near complete remission (nCR) or very good partial remission (VGPR) prior to HD/ASCT treatment. The median age at HD/ASCT therapy was 61 (range 41–75) years. Melphalan dose modifications were performed for 2 (1%) patients. After HD/ASCT therapy, the number of patients who achieved CR, nCR or VGPR increased to 111 (74%).

Other than the number of reinfused CD34+ cells (given by the definition of the groups), no statistically significant differences were found between groups 1 (3–4 × 10^6^ CD34+ cells/kg bw) and 2 (2–2.5 × 10^6^ CD34+ cells/kg bw) with regard to HD/ASCT treatment. Details of the HD/ASCT therapy for the overall cohort and the subgroups are summarized in Table 2.

**Table 2:** High-dose chemotherapy/ASCT

Parameter	Overall cohort	Group 1 (3–4 × 10^**6**^ CD34+ cells /kg bw)	Group 2 (2–2.5 × 10^**6**^ CD34+ cells /kg bw)	***P*** value Group 1 vs. 2	Group 3 (< 2 × 10^**6**^ CD34+ cells /kg bw)
**ASCTs analyzed, n**	148	86	53	/	9
**Sequential ABSCTs, n (%)**				/	
First	142 (96)	86 (100)	53 (100)		3 (33)
Second	5 (3)	0 (0)	0 (0)		5 (56)
Third	1 (1)	0 (0)	0 (0)		1 (11)
**Remission pre ABSCT, n (%)**				0.168^a^	
CR	2 (1)	2 (2)	0 (0)		0 (0)
nCR	38 (26)	28 (33)	9 (17)		1 (11)
VGPR	48 (32)	26 (30)	21 (40)		1 (11)
PR	45 (30)	21 (24)	18 (34)		6 (67)
MR	4 (3)	3 (3)	1 (2)		0 (0)
SD	1 (1)	0 (0)	1 (2)		0 (0)
PD	6 (4)	2 (2)	3 (6)		1 (11)
NA	4 (3)	4 (5)	0 (0)		0 (0)
**Median age at ASCT, years (range)**	61 (41–75)	61 (44–73)	62 (41–72)	0.886	60 (50–75)
**Transplanted PBSCs**					
Median transplanted CD34+ cells ×10^6^/kg (range)	3.2 (1.7–4.0)	3.6 (3.0–4.0)	2.3 (2.0–2.5)	**< 0.001**	1.9 (1.7–1.99)
Median vitality, % (range)	79 (53–93)	76 (53–93)	81 (58–93)	0.012	80 (66–93)
**HD chemotherapy, n (%)**				/	
Melphalan 2 × 100 mg/m^2^	146 (99)	85 (99)	53 (100)		8 (89)
Dose reduction	2 (1)	1 (1)	0 (0)		1 (11)
**Remission post ASCT, n (%)**				0.316^b^	
CR	15 (10)	11 (13)	4 (8)		0 (0)
nCR	42 (28)	30 (35)	11 (21)		1 (11)
VGPR	54 (36)	27 (31)	22 (42)		5 (56)
PR	25 (17)	11 (13)	12 (23)		2 (22)
MR	5 (3)	3 (3)	2 (4)		0 (0)
SD	1 (1)	1 (1)	0 (0)		0 (0)
PD	2 (1)	2 (2)	0 (0)		0 (0)
NA	4 (3)	1 (1)	2 (4)		1 (11)

^a/b^CR/nCR/VGPR versus PR/MR/SD/PD.

*ASCT* autologous blood stem cell transplantation; *CR* complete remission; *HD* high-dose; *MR* minimal response; *NA* not available; *nCR* near complete remission; *PD* progressive disease; *PR* partial remission; *SD* stable disease; *VGPR* very good partial remission; vs., versus

### Hematopoietic reconstitution according to the number of transplanted CD34+ cells

All patients reached hematopoietic reconstitution after HD/ASCT treatment, even those who received < 2 × 10^6^ CD34+ cells/kg bw (group 3). Since the number of patients in group 3 (< 2 × 10^6^ CD34+ cells/kg bw) was very low (*n* = 9), statistical comparisons were performed between groups 1 (3–4 × 10^6^ CD34+ cells/kg bw) and 2 (2–2.5 × 10^6^ CD34+ cells/kg bw) only (Table 3).

**Table 3:** Hematopoietic reconstitution after high-dose chemotherapy/ASCT by number of transplanted CD34+ cells

Parameter	Overall cohort	Group 1 (3–4 × 10^**6**^ CD34+ cells /kg bw)	Group 2 (2–2.5 × 10^**6**^ CD34+ cells /kg bw)	***P*** value Group 1 vs. 2	Group 3 (< 2 × 10^**6**^ CD34+ cells /kg bw)
**ASCTs analyzed, n**	148	86	53		9
**G-CSF support, n (%)**				0.271	
Yes	62 (42)	34 (40)	26 (49)		2 (22)
No	86 (58)	52 (60)	27 (51)		7 (78)
**Leukocyte reconstitution**				0.393	
n available	144	82	53		9
Days to L ≥ 1.0 × 10^9^/L	12 (9–24)	12 (9–23)	12 (10–24)		12 (9–16)
**Neutrophil reconstitution**				/	
n available	42	17	23		2
Days to *N* ≥ 0.5 × 10^9^/L	14 (9–19)	14 (9–19)	13 (10–18)		13 (11–14)
**Aplasia**				0.513	
n available	116	62	46		8
Days in aplasia	9 (4–20)	9 (4–19)	8 (5–20)		9 (5–13)
**Platelet reconstitution**				**< 0.001**	
n available	144	85	51		8
Days to platelets ≥20 × 10^9^/L	12 (9–21)	11 (9–16)	13 (10–21)		13 (9–19)
n available	81	55	23	**0.001**	3
Days to platelets ≥50 × 10^9^/L	14 (10–22)	14 (10–18)	14 (13–22)		15 (13–18)

If not otherwise indicated, the data are presented as the median (range)

*ASCT* autologous blood stem cell transplantation; *G-CSF* granulocyte-colony stimulating factor; *L* leukocytes, *NA* not available; *N* neutrophils; vs., versus

**Fig. 1:**
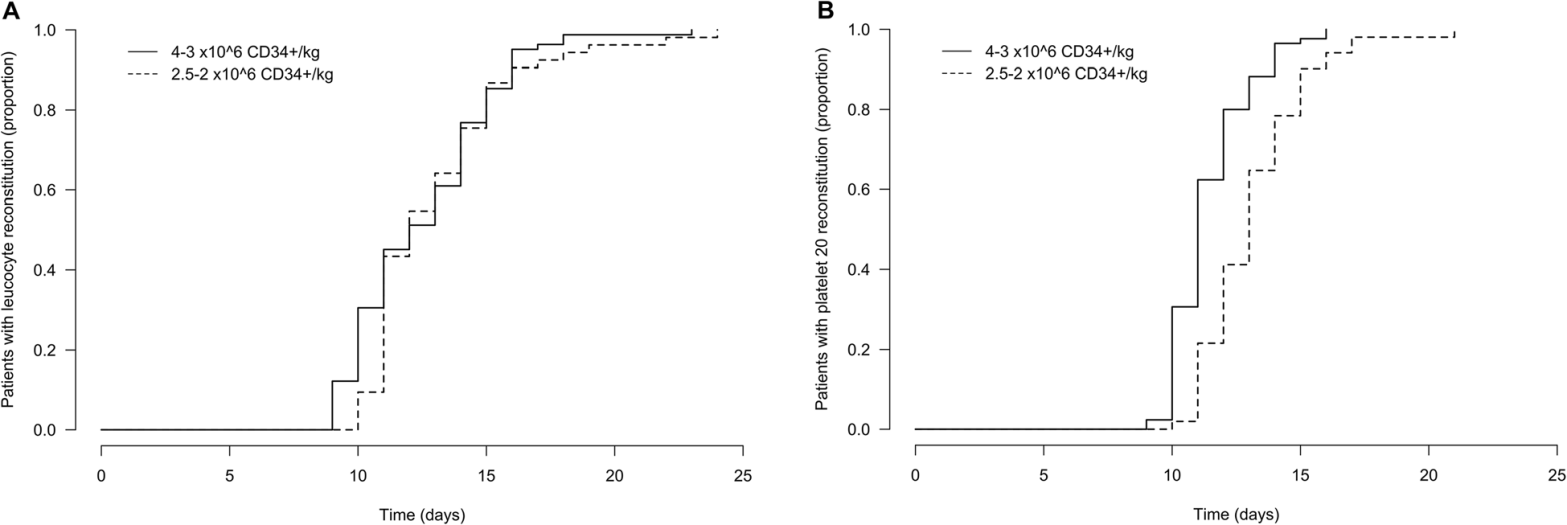
Hematopoietic reconstitution after HD/ASCT by the number of reinfused CD34+ cells. The relative number of patients with leukocyte recovery ≥1.0 × 10^9^/L (**a**) and platelet recovery ≥20 × 10^9^/L (**b**) is shown. The results are grouped according to the number of reinfused CD34+ cells (3–4 versus 2–2.5 × 10^6^ CD34+ cells/kg bw)

The median time to achieve leukocytes ≥1.0 × 10^9^/L after PBSC reinfusion was 12 days in all groups and ranged between 9 and 23 days, 10–24 days and 9–16 days in groups 1, 2 and 3, respectively. No statistically significant difference in time to leukocyte engraftment was observed between groups 1 and 2 (Fig. 1**A**, *p* = 0.393). The median duration of aplasia was 9 (range 4–19), 8 (range 5–20) and 9 (5–13) days for groups 1, 2 and 3, respectively, and no statistically significant differences were found between groups 1 and 2.

Neutrophil reconstitution was evaluated in a small proportion of patients (n_group1_ = 17, n_group2_ = 23, n_group3_ = 2) only. The median time from ASCT to neutrophil recovery was 14 (range 9–19), 13 (range 10–18) and 13 (11–14) days for groups 1, 2 and 3, respectively.

The median duration to platelet recovery ≥20 × 10^9^/L was 11 (range 9–16), 13 (range 10–21) and 13 (9–19) days for groups 1, 2 and 3, respectively. Patients who received a high number of CD34+ cells (3–4 × 10^6^ CD34+ cells/kg bw, group 1) showed a faster platelet ≥20 × 10^9^/L recovery than patients who received a low number of reinfused CD34+ cells (2–2.5 × 10^6^ CD34+ cells/kg bw, group 2) (Fig. 1**B**, *p* < 0.001).

Data on platelet recovery ≥50 × 10^9^/L were available in a smaller proportion of patients (n_group1_ = 55, n_group2_ = 23, n_group3_ = 3) only. The median duration to platelet recovery ≥50 × 10^9^/L was 14 (range 10–18), 14 (range 13–22) and 15 (13–18) days for groups 1, 2 and 3, respectively. Similar to platelet reconstitution ≥20 × 10^9^/L, the log-rank comparison revealed a significantly faster platelet recovery ≥50 × 10^9^/L in patients who received a high number of CD34+ cells than patients who received a low number of CD34+ cells (*p* = 0.001).

**Fig. 2:**
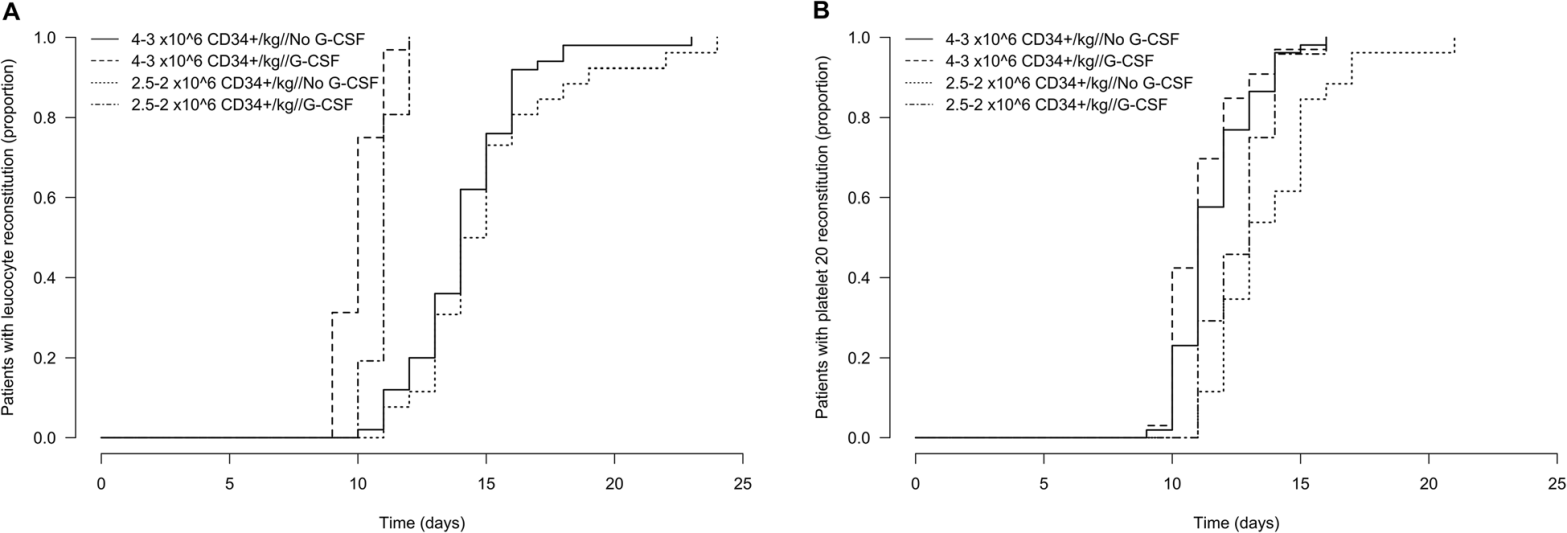
Hematopoietic reconstitution after HD/ASCT by the number of reinfused CD34+ cells and by G-CSF support status. The relative number of patients with leukocyte recovery ≥1.0 × 10^9^/L (**a**) and platelet recovery ≥20 × 10^9^/L (**b**) is shown. The results are grouped according to the number of reinfused CD34+ cells (3–4 versus 2–2.5 × 10^6^ CD34+ cells/kg bw) and G-CSF support status

Overall, the univariate analysis revealed an association between a higher number of reinfused CD34+ cells and fast platelet recovery after ASCT. But, this effect was not evident for leukocyte reconstitution. As a proportion of the analyzed patients (*n* = 62, 42%) received G-CSF support after ASCT, we hypothesized that G-CSF administration might significantly accelerate leukocyte reconstitution and mask the influence of the number of reinfused CD34+ cells. A subgroup analysis based on the number of reinfused CD34+ cells and G-CSF support status showed that the median time to leukocyte reconstitution was significantly shortened by G-CSF support from 14 to 10 days in the 3–4 × 10^6^ CD34+ cells/kg bw group and from 14 to 11 days in the 2–2.5 × 10^6^ CD34+ cells/kg bw group (*p* < 0.001, respectively; Fig. 2**A**). G-CSF administration significantly shortened the time to platelet recovery ≥20 × 10^9^/L in the 2–2.5 × 10^6^ CD34+ cells/kg bw group (*p* = 0.020) but not in the 3–4 × 10^6^ CD34+ cells/kg bw group (*p* = 0.200, Fig. 2**B**). No statistically significant differences in time to platelet recovery ≥50 × 10^9^/L were observed with regard to G-CSF administration either in the 3–4 × 10^6^ CD34+ cells/kg bw group (*p* = 0.800) or in the 2–2.5 × 10^6^ CD34+ cells/kg bw group (p = 0.200).

In the multivariate analysis, neither age at ASCT nor remission status pre-ASCT affected the duration of hematopoietic reconstitution. However, the number of reinfused CD34+ cells significantly influenced the duration until hematopoietic recovery. A low number of reinfused CD34+ cells at ASCT was associated with significantly prolonged time until leukocyte reconstitution ≥1.0 × 10^9^/L (*p* = 0.010) and platelet recovery ≥20 × 10^9^/L (< 0.001) and ≥ 50 × 10^9^/L (*p* = 0.003). As indicated by the univariate analysis, G-CSF support after ASCT significantly accelerated leukocyte reconstitution (*p* < 0.001) but not platelet reconstitution. The results of the multivariate analysis including the hazard ratio (HR) and 95% confidence interval (CI_95%_) are given in Table 4.

**Table 4:** Hematopoietic reconstitution - multivariate analysis

Parameter	Leukocyte reconstitution (≥1.0 × 10^**9**^/L)		Aplasia		Platelet reconstitution (≥20 × 10^**9**^/L)		Platelet reconstitution (≥50 × 10^**9**^/L)	
**n analyzed**	130		103		131		76	
	**HR (CI**_**95**_**)**	**P value**	**HR (CI**_**95**_**)**	**P value**	**HR (CI**_**95**_**)**	**P value**	**HR (CI**_**95**_**)**	**P value**
**Age at ABSCT (≤60 vs. > 60 years)**	1.038 (0.730–1.476)	0.837	1.101 (0.725–1.669)	0.652	1.086 (0.762–1.547)	0.649	0.841 (0.529–1.336)	0.463
**Remission pre ABSCT (CR/nCR/VGPR vs. PR/MR/SD/PD)**	0.989 (0.681–1.436)	0.952	1.129 (0.735–1.734)	0.581	1.098 (0.755–1.598)	0.625	1.060 (0.650–1.729)	0.815
**G-CSF support (no vs. yes)**	16.742 (8.514–32.923)	**< 0.001**	9.634 (5.425–17.107)	**< 0.001**	1.365 (0.951–1.958)	0.091	1.084 (0.655–1.794)	0.753
**CD34+ cells/kg bw transplanted (3–4 vs. 2–2.5 × 10**^**6**^**)**	0.607 (0.416–0.885)	**0.010**	0.573 (0.375–0.875)	**0.010**	0.438 (0.299–0.642)	**< 0.001**	0.442 (0.258–0.755)	**0.003**

*ASCT* autologous blood stem cell transplantation; *CI* confidence interval; *CR* complete remission; *HR* hazard ratio; *G-CSF* granulocyte-colony stimulating factor; *MR* minimal response; *nCR* near complete remission; *PD* progressive disease; *PR* partial remission; *SD* stable disease; *VGPR* very good partial remission; vs., versus

No severe adverse events were observed during or after the considered HD/ASCT in the analyzed cohort.

## Discussion

We retrospectively analyzed the short-term hematopoietic reconstitution in MM patients who received a low-dose PBSC graft after HD chemotherapy with melphalan.

A small cohort of MM patients (*n* = 9) received a very low (< 2.0 × 10^6^/kg bw) number of CD34+ cells after HD chemotherapy. These numbers might be an insufficient PBSC graft, as defined by the current national guidelines and international agreements [14, 30]. However, despite the low CD34+ cell count of the transplant all of the patients in group 3 reached hematopoietic reconstitution. Although not assessable by comparative statistics due to low patient numbers, the median time until leukocyte recovery ≥1.0 × 10^9^/L (12 days) and platelet recovery ≥20 × 10^9^/L (13 days) in patients who received low numbers of CD34+ cells was similar or even identical to that of patients who received PBSC grafts with high numbers of CD34+ cells. This is in line with the results of earlier studies that demonstrated successful hematopoietic reconstitution in MM patients who received 1.0–2.0 × 10^6^ CD34+ cells/kg as autologous grafts after HD therapy [31, 32]. Nevertheless these and further studies also demonstrated that the use of high CD34+ cell doses reduces the time until hematopoietic recovery and lowers the risk of graft failure [33]. An analysis of engraftment kinetics after myeloablative chemotherapy additionally showed a clear dose-response relationship between the number of CD34+ cells infused and neutrophil and platelet engraftment. Although a minimal threshold CD34+ cell dose could not be defined, ≥5.0 × 10^6^ CD34+ cells/kg appeared to be optimal [34]. Furthermore in allogeneic T cell-depleted bone marrow transplants it has been reported that CD34+ cell dose was the only variable significantly associated with treatment-related mortality, primarily due to infections and cytopenia and therefore higher CD34+ cell doses may improve outcome in engrafting [35].

All MM patients who received low-dose (2–2.5 × 10^6^ CD34+ cells/kg bw) PBSC grafts in this analysis also showed successful hematopoietic recovery after HD melphalan treatment. Due to the large number of evaluated patients, the cohort was accessible to comparative statistics. Therefore, MM patients who received 3–4 × 10^6^ CD34+ cells/kg bw at ASCT were chosen as the comparator group. As previously reported, this number represents the median reference value of reinfused CD34+ cells at our institution [20, 21]. Both cohorts had similar age but not sex distributions. Importantly, the type of induction treatment and mobilization therapy was similar in both groups, and no statistically significant differences were identified with regard to remission status prior to ASCT. Therefore, the comparison between groups is based on highly homogeneous cohorts, which, in addition to relatively high case numbers, represents a major strength of the current analysis.

The time to leukocyte, neutrophil and platelet recovery after HD/ASCT treatment observed in patients who received 3–4 × 10^6^ CD34+ cells/kg bw at ASCT was similar to that previously described in MM patients. In particular, Gerzt et al. reported a median 15 days until neutrophil and platelet recovery ≥50 × 10^9^/L, which is in line with our findings [36].

As revealed by multivariate analysis, reinfusion of lower numbers of CD34+ cells (2–2.5 compared to 3–4 × 10^6^ CD34+ cells/kg bw) was associated with significantly prolonged time to leukocyte recovery ≥1.0 × 10^9^/L and platelet recovery ≥20 × 10^9^/L and ≥ 50 × 10^9^/L. This is in line with the findings of previous reports that emphasized the positive correlation between the CD34+ cell dose and time to hematopoietic reconstitution [30, 37, 38]. Remarkably a further study showed that patients who received lower stem cell doses had an increased risk of > 3 days of absolute neutropenia, compared to patients who received higher stem cell infusions, while at a median follow-up of 51 months, there was no difference in survival between patients with absolute neutropenia > 3 days versus patients with absolute neutropenia for ≤3 days [39]. On the other hand, it was reported that for older MM patients undergoing HD chemotherapy and ASCT infusion of higher stem cell doses did not yield a reduction in symptom burden or engraftment time in the first weeks after ASCT [40]. Also in accordance to this study multiple, fractionated stem cell infusions (days 0, + 2, + 4, + 6) following HD melphalan did not enhance engraftment kinetics or significantly alter MM patients’ clinical course following ASCT [41].

G-CSF support after HD/ASCT treatment significantly shortened the time until leukocyte recovery ≥1.0 × 10^9^/L but not until platelet recovery. These findings are consistent with previous studies, demonstrating that leukocyte and neutrophil engraftment after autologous progenitor cell transplantation can be accelerated by G-CSF support [42–44]. As reported by several studies a single dose of pegfilgrastim is a safe and efficacious alternative to daily injections of filgrastim while patients who received pegfilgrastim showed faster engraftment, lower incidence of febrile neutropenia and a shorter hospitalization [42, 45].

As demonstrated by multivariate analysis, G-CSF support accelerates leukocyte engraftment to a much higher extent than a large reinfusion dose of CD34+ cells (HR 16.742 versus 0.607). To the best of our knowledge, this is the first analysis to evaluate the mutual effect of G-CSF administration and CD34+ cell dose on hematopoietic recovery after ASCT.

No severe adverse events were observed during or after the considered HD/ASCT in the analyzed cohort of all 148 MM patients. Of note, during the analysis period we recorded one heavily pretreated 60-year-old female patient who presented with a severe adverse event (Pneumocystis jiroveci pneumonia) after a second HD/ASCT receiving a low-dose PBSC graft (2.15 × 10^6^ CD34+ cells/kg bw). However, this patient did not meet the inclusion criteria of the current analysis (not first HD/ASCT) and was therefore not evaluated in the study cohort. Remarkably in contrast to that finding, none of the intensely pretreated patients in group 3 presented with a severe adverse event (no severe infections or transfer to intensive care unit was reported) after autologous transplant of a very low PBSC graft.

So far there is no reported clinical experience in the reinfusion of PBSC grafts below the minimum of 2.0 × 10^6^ CD34+ cells/kg bw defined by international guidelines. Yet there are many factors, such as higher age of MM patients, prior extensive chemotherapy or radiation therapy, which are associated with poor PBSC mobilization. Triplet regimens that include the immunomodulatory agent lenalidomide have emerged as standard-of-care induction therapy in transplant-eligible patients with MM. However, lenalidomide has been reported to have an adverse effect on PBSC collection [46–48]. A correlation between the length of lenalidomide therapy and decrease in PBSC yield has been reported by different groups. Up to four cycles of lenalidomide exposure may have minimal negative impact on PBSC collection and Plerixafor may overcome these negative effects [49, 50]. Nevertheless, it may be challenging to achieve the target PBSC yield after lenalidomide-containing regimens and it may result in low-dose grafts with CD34+ cell doses < 2.0 × 10^6^/kg bw. Reporting the few available patients, we aimed to exemplarily demonstrate an adequate engraftment of PBSC grafts with CD34+ cell count below the internationally accepted threshold of 2.0 × 10^6^/kg bw and therefore to encourage other centers to perform ASCTs with very low dose PBSC grafts. This is particularly of outstanding importance in a clinical setting when a HD chemotherapy and ASCT represent a therapeutic option but an additional PBSC collection is not feasible.

## Conclusion

In conclusion, our study demonstrates that quantitative and timely sufficient hematopoietic reconstitution is achievable upon reinfusion of low-dose PBSC grafts after HD therapy in MM patients. Further evaluation is required to confirm adequate hematopoietic engraftment in more MM patients who receive very low dose PBSC grafts with < 2.0 × 10^6^ CD34+ cells/kg bw after HD chemotherapy. While the impact of the CD34+ cell dose is significant but clinically marginal, G-CSF support substantially accelerates the time until leukocyte recovery.

## Data Availability

The datasets generated and/or analyzed during the current study are not publicly available due to current data protection directive but are available from the corresponding author on reasonable request within 6 months after publication of the manuscript.

## Abbreviations

ASCT: Autologous blood stem cell transplantation
bw: Body weight
CAD: Cyclophosphamide, doxorubicin, dexamethasone
CI: Confidence interval
CR: complete remission
DMSO: Cryosure-D dimethyl sulfoxide
G-CSF: Granulocyte-colony-stimulating factor
HD: High-dose
HR: Hazard ratio
ISS: International Staging System
L: Liter
LP: Leukapheresis
MM: Multiple myeloma
NC: Nucleated cell
nCR: Near complete remission
PBSC: Peripheral blood stem cell
p.o.: Per os
PR: Partial remission
VCD: Bortezomib, cyclophosphamide, dexamethasone
VGPR: Very good partial remission
VRD: Bortezomib, lenalidomide, dexamethasone
